# Effects of a Specific Trunk and Shoulder Strength Training Program on Throwing Velocity and Accuracy: A Study Among Hungarian First-League Female Handball Players

**DOI:** 10.3390/sports12110296

**Published:** 2024-10-31

**Authors:** Gréta Csilla Sinka, Ágnes Mayer, András Tállay, Miklós Tátrai, Lilla Tábori, Eszter Papp, Attila Pavlik

**Affiliations:** 1 Doctoral School, Semmelweis University, 1085 Budapest, Hungary; 2 Faculty of Physiotherapy, Semmelweis University, 1088 Budapest, Hungary; 3 Faculty of Sportsmedicine, Semmelweis University, 1122 Budapest, Hungary; 4 Department of Traumatology, St. John Hospital, 1125 Budapest, Hungary; 5 Department of Sports Surgery, National Institute for Sports Medicine, 1113 Budapest, Hungary

**Keywords:** shoulder joint instability, handball, speed, accuracy, trunk stability

## Abstract

The aim of the study was to record shoulder and trunk stability of elite female handball players and to investigate their effect on throwing velocity and accuracy. 151 female handball players (9 teams in total) from the Hungarian first league participated in a mid-season conditional survey. The study included tests of trunk and shoulder stability, and measurements of factors affecting shoulder stability, as well as throwing speed and throwing accuracy. After the conditional survey, 18 players were selected for the intervention group (IG) and 18 players for the control group (CG). Significant relationships were found between the medial stability of the shoulder joint (Y-balance) and throwing accuracy (upper right corner) (r = 0.766; *p* < 0.001), throwing accuracy (upper left corner) (r = 0.729; *p* < 0.001), total throwing accuracy (r = 0.907; *p* < 0.001). The IG showed significant improvement in trunk stability (*p* < 0.001), shoulder joint stability (*p* < 0.001), throwing velocity (*p* < 0.001), and throwing accuracy (*p* = 0.002), compared to the CG. These findings support the idea that trunk and shoulder stability are related to throwing performance of female handball players. Measuring and training these aforementioned factors, particularly medial shoulder stability, may be a valuable adjunct to improving throwing accuracy and throwing velocity.

## 1. Introduction

Previous studies have identified a number of factors that can lead to the development of shoulder joint injuries [1,2,3,4]. The identified risk factors mostly affect the normal biomechanics of the shoulder joint [5,6,7,8], such as decreased internal rotational passive range of movement (IR PROM) [9,10], external and internal rotator muscle (ER/IR) ratio deviation [9,11], dynamic stabilizing muscle dysfunction which affect shoulder stability and can be assumed to affect shooting performance as well [8], the tightness of the posterior part of the capsule [12] and the postero-superior impingement caused by the repetitive throwing movements [13,14]. According to some studies, up to 19–36% of handball players reported shoulder problems at the beginning of the season [15] and 28% during the season [6,9] regardless of gender. Most studies have focused on the identification of risk factors and rehabilitation protocols, because of the high prevalence and importance [2,3,4,12]. It turned out that several of the risk factors found in the background of injuries affect shoulder stability—such as PROM, ER/IR ratio, scapular dyskinesis—and as a result sports science gained a greater insight into the factors influencing shoulder joint stability.

Despite the fact that trunk and shoulder stability is often mentioned as an important determinant of better sports performance, to our knowledge, there are a very few study that supports this notion in the case of the throwing performance of female handball players.

The correlation between several shoulder joint stability tests, such as the ER/IR ratio, PROM, CKCUEST (Closed Kinetic Chain Upper Extremity Stability Test), Upper Quarter Y-balance Test (YBT-UQ) and the injury rate has already been investigated [3,7,16,17], but their association with shooting performance has not been cleared.

The results of Bauer et al.’s (2020) research showed that the YBT-UQ does not show a correlation between shoulder stability and shooting performance, but it can predict an increase in the risk of shoulder joint injuries [16]. Clarsen et al. (2014) discovered, that the deviation from the normal value of the ER/IR ratio, especially if manifests itself in the reduction of the strength of the external rotator muscles, increases the chance of shoulder injuries [6]. Later Mascarin et al. (2017) highlighted that shoulder stabilization exercises with elastic bands are necessary to improve (ER/IR) ratios and shooting speed [18]. Although in the aforementioned manuscript could not demonstrate a correlation between muscle strength ratio and shooting speed, it concluded as a limitation that the 6-week program may not have been long enough, and it would be worth investigating the relationship further [19]. Te decrease in IR PROM has been identified as a possible risk factor for shoulder injuries, due to the connective soft tissue stiffness [20].

Although several studies have focused on examining the difference in performance between athletes with shoulder joint problems and their healthy counterparts at YBT-UQ and CKCUES tests, the relationship between these tests and sport-specific skills remains unclear.

Therefore, the primary purpose of the present paper was to investigate the impact of shoulder and trunk stability on shooting skills, such as shooting accuracy and shooting velocity. The secondary purpose of this study was to examine whether throwing performance can be improve with an intervention program designed to enhance shoulder strength, mobility and stability. It was hypothesized that a better trunk and shoulder stability leads to better throwing performance.

## 2. Materials and Methods

### 2.1. Study Design

This was a quantitative, longitudinal, prospective study that included the results of a baseline assessment and an intervention study.

### 2.2. Participants

**Figure 1:**
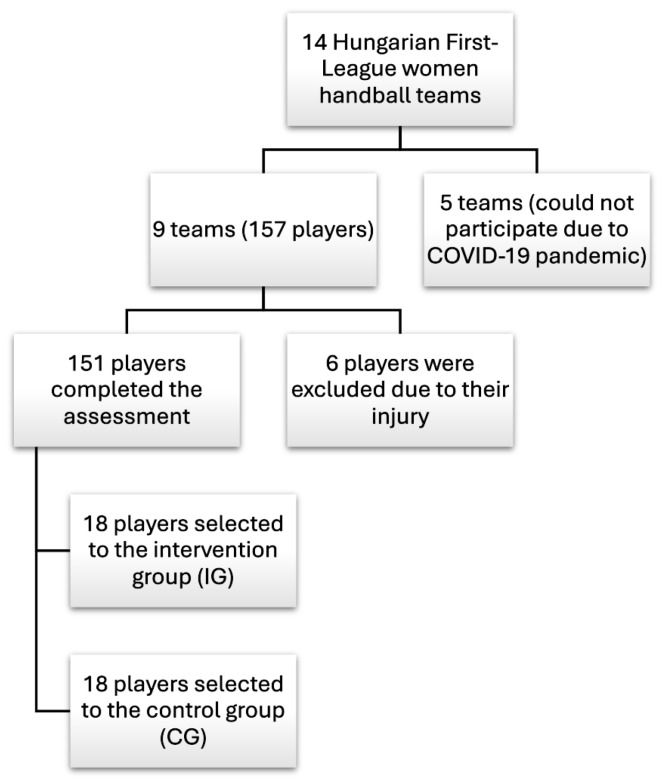
Team recruitment and drop-out.

The assessment period was six months (from October 2019 to March 2020) of the competitive period of the season. Of the 14 teams in the Hungarian Handball Women’s First-League, 9 teams (157 players) were involved in the investigation (Figure 1).

In order to verify measurement results, there were no match situation and stabilization training sessions in the 2 days before the survey, and no training sessions for 24 h before the assessment. The study was approved by he Hungarian Handball Association and the Regional Scientific and Research Ethics Committee of the St. Emeric Hospital and St. John Hospital. For ethical compliance, we requested the permission of the Medical Ethics Committee to conduct the research, which is included in Declaration of Interest Statement file (license number 01-18068 i12978, -12979). The faces of the athletes were obscured in the photographs, and the data were not disclosed to third parties.

### 2.3. Equity, Diversity, and Inclusion Statement

The author group was gender-balanced and consisted of junior, mid-career and senior researchers from different disciplines; however, all members of the author group were from the same country. The given study focused on women handball players of the Hungarian Premier League level, excluding women and girls playing handball at lower levels and those who were playing abroad during the competitive season.

### 2.4. Recruitment and Inclusion/Exclusion Criteria

Inclusion criteria were that the player attended at least 7 training sessions per week, played in the premier league, and agreed to be tested. Exclusion criteria were that the player had a defined history of traumatic shoulder pain, shoulder surgery within one year, and acute inflammation (based on physical examination and interviews with the medical staff). Of the 157 players, 6 were excluded at the baseline due to injury, or acute inflammation.

After the conditional assessment, 18 players were randomly selected from the teams located in Budapest for both the intervention and control groups, which included players from first-league teams in Budapest, and the same number of players from these teams were selected for the control group. The reason for this was controllability. Not all teams had a full-time physiotherapist, and the teams were scattered across the country, and given the overall duration of the program, the research group could not have assigned a person to each group to provide full supervision. This solution provided the greatest controllability, as the majority of the research team members were based in Budapest. If at least 80% of the program was not completed, the athlete was excluded from the intervention group.

### 2.5. Compliance

The participation of the intervention group in the program was observed. 1 person could not attend the session 3 times, 6 people 2 times, 8 people 1 time.

### 2.6. Baseline Testing

In order to control the measurement results, there was no match situation and stabilization training in the 2 days before the survey, and there was no training for 24 h before the assessment.

#### 2.6.1. Assessment of Shoulder Stability

**Figure 2:**
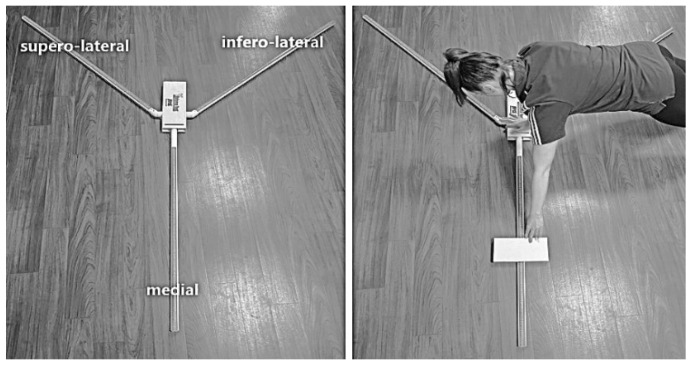
Y balance test and a medial slide.

Shoulder joint stability was examined using the YBT-UQ [17] and CKCUES [21] tests. The Y-balance test was performed with the handball players reaching in the medial, supero-lateral, and infero-lateral directions, while maintaining the high-plank position (Figure 2).

#### 2.6.2. Assessment of Trunk Stability

**Figure 3:**
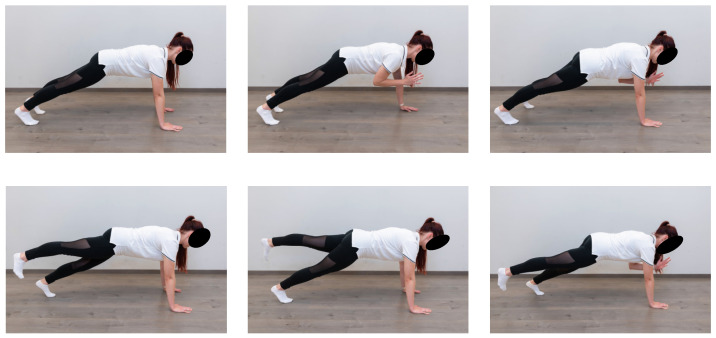

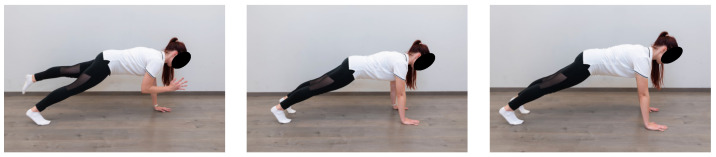
Core-max test shown by one of the authors.

Trunk stability was examined using the 4-point plank [19], side plank, and Core-max [22] tests. During the Core-max test, the athletes were required to hold themselves in a high-plank position for one minute and then change positions every 15 s (Figure 3).

#### 2.6.3. Assessment of Shoulder ER/IR ROM and ER/IR Strength

Passive shoulder external and internal rotation range of motion (PROM) was measured using a goniometer in the supine position, with a 90° abducted shoulder joint position, and a bent elbow in a neutral position [10,20].

The isometric strength of the external and internal rotator muscles of the shoulder was measured in the same position as PROM, in the middle of the rotational range of motion with isometric muscle contraction, using a digital hand-held dynamometer (ErgoFET, Hoggan Health Industries, Salt Lake City, UT, USA) [20,23].

#### 2.6.4. Assessment of Shooting Speed and Shooting Accuracy

In the throwing speed [24] test, the players were 7 m from the goal line and positioned in the middle of the cage. The formula v = s/t was used to calculate the velocity. The players had to throw with their elbow at the level or above the level of shoulder height [16]. If the athlete did not perform the described way the trial was repeated.

Throwing accuracy [16] was measured with the athletes standing 7 m from the goal line and positioned in the middle of the cage. At the whistle, the players were randomly asked to hit the triangle in the upper right and left corner of the cage as many times as possible out of ten shots (60 cm long horizontally, 40 cm long vertically and 72 cm long diagonally; held by bungeer cords).

Supplementary Materials contains a detailed description of the interventions.

During the assessment, a given test was always measured by the same person, thereby eliminating measurement deviations between examiners (inter-examiner reliability). Table 1 shows the test reliability and validity.

### 2.7. Training Program

A three-month, one-hour training program was performed twice a week, based on strengthening the trunk, periscapular, rotator cuff muscles, especially the external rotators, and shoulder stabilizing muscles of the players. The development of lumbar motor control was important too. Plyometric exercises were performed to improve the strength of external rotator muscles. During the exercises, the Isotonic combination techniques, stretching and re-stretching stimuli were also used many times to facilitate as many motor units as possible. The introduction of proprioceptive training and sport-specific exercise sequences adapted to the specialties of handball was essential. Reconstructing these situations was key to the construction of the program because the shoulder joint needed to be prepared for the strength typical of the sport. Training sessions were concluded by a twenty-minute stretching session each time, in which contract-relax and static stretching exercises were performed (Supplementary Materials).

Post intervention testing

Both the intervention and control groups were re-tested immediately after the end of the training program. Similar to the baseline test, there was no match situation and stabilization training 2 days before the survey, and no training for 24 h before the assessment.

### 2.8. Statistics

Data were entered into Microsoft Office Excel 2019, where descriptive statistics (mean, standard deviation, and frequency) were calculated. Mathematical statistical calculations were performed using the IBM SPSS 24.0 system, Wilcoxon test or Mann-Whitney test depending on the distribution. Pearson’s correlation was applied to analyze the relationship between trunk stability, shoulder stability, and throwing parameters. A paired *t*-test was used to examine the effectiveness of the training program. The results were considered significant at *p* ≤ 0.05.

## 3. Results

### 3.1. Participants

The participant’s were 23.5 ± 4.7 years old on average, their average body weight was 70.6 ± 8.2 kg, and their average height 175.3 ± 6.2 cm. In terms of the number of years spent in handball, the average was 14.1 ± 4.1 years, with a minimum of 6 years and a maximum of 18 years.

### 3.2. Baseline Testing

There was a significant positive association between the medial stability of the shoulder joint (Y-balance) and throwing accuracy (upper right corner) (r = 0.766; *p* < 0.001), throwing accuracy (upper left corner) (r = 0.729; *p* < 0.001), total throwing accuracy (r = 0.907; *p* < 0.001). There was a positive association between the supero-lateral stability of the shoulder joint (Y-balance) and throwing accuracy (upper right corner) (r = 0.498; *p* < 0.001), sthrowinghooting accuracy (upper left corner) (r = 0.637; *p* < 0.001), total throwing accuracy (r = 0.694; *p* < 0.001); as well as between the supero-lateral stability of the shoulder joint (Y-balance) and the throwing velocity (r = 0.231; *p* = 0.004).

A correlation was detected between the isometric strength of the external rotators (ErgoFet) and the infero-lateral stability of the shoulder joint (Y-balance) (r = 0.234; *p* = 0.004).

There was a positive relationship between trunk stability (4-point plank) and total throwing accuracy (r = 0.234; *p* = 0.004), throwing accuracy (left upper corner) (r = 0.223; *p* = 0.006), and throwing velocity (r = 0.215; *p* = 0.008). Between the stability of the side chain of the trunk (dominant plank) and the total throwing accuracy (r = 0.261; *p* = 0.001), throwing accuracy (upper right corner) (r = 0.180; *p* = 0.027), throwing accuracy (left upper corner) (r = 0.247; *p* = 0.002) there was a positive association too.

All correlation results are shown in Table 2**.**

### 3.3. Training Program Results

In the IG the participant’s were 21.9 ± 4.2 years old on average, their average body weight was 70.8 ± 7.0 kg, and their average height 170.3 ± 6.8 cm. In the CG the participant’s were 22.2 ± 2.8 years old on average, their average body weight was 73.4 ± 5.6 kg, and their average height 172.3 ± 7.2 cm. After the training program, there were significant improvements in the IG shoulder stability (CKCUES test), shooting velocity, 4-point plank, and dominant side plank (*p* < 0.001). Significant changes were also detected in the Core-max test (*p* = 0.04) in the IG. Compared to the significant changes in the IG, only 4-point plank (*p* = 0.002) and dominant side plank (*p* = 0.01) showed significant changes in the CG.

**Figure 4:**
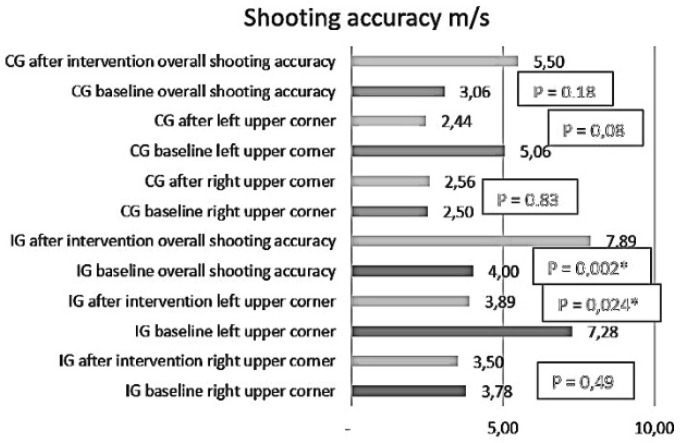
The changes in throwing accuracy in the IG and the CG.

Figure 4 shows changes in shooting accuracy in the IG and the CG.

Table 3 shows changes due to the training program in the IG and in the CG after three months.

## 4. Discussion

The present study shows that the sport-specific skills of handball players are related to the shoulder and trunk stability. Furthermore, it can be stated that different directions of shoulder stability, especially medial and supero-lateral direction, affect throwing accuracy. In particular, players with better Y-balance results, higher CKCUEST results, increased trunk stability, and normal (or near normal) IR PROM [6] were more likely to have a better performance. A better external rotator muscle strength [6,11] leads to a better shoulder stability in infero-lateral direction. Perhaps improved trunk and shoulder stability provides a good basis for mechanically efficient throwing performance. Probably the enchanced performance detected in the present paper is consistent with other studies focusing on strengthening [18,19].

### 4.1. Shoulder Stability and Shooting Performance

The throwing arm must be both mobile and stable, which requires special adaptations. Accordingly, the test method must also have to be multi-component. A possible and validated method for examining the stability and mobility of the shoulder joint is the YBT-UQ test [16].

In Jha P. et al.’s (2022) study a 5 weeks long core-stabilizing program made 19% improvement in YBT-UQ results and 35% improvement in throwing accuracy results [25]. In addition these results, Bauer J. et al. (2020) investigated the correlation between the YBT-UQ test for shoulder joint stability and shooting performance [16]. They did not find any significant correlation with the throwing performance, except for the supero-lateral direction. In the present study of female handball players, a significant relationship was found between Y-balance test scores in different directions and tests scores for shooting accuracy and shooting velocity.

The difference in the results may be related to the number of participants. No significant correlation was found in the research with lower number of participants in the different gender and age groups, contrary to those involving more than 70 people, where the numbers stood out better.

### 4.2. Trunk Stability and Shooting Performance

Previous reviews and meta-analyses have focused on trunk stability and sport-specific performance [26]. They concluded, if these variables are analyzed, as has been done in Rodriguez-Perea Á. et al.’s meta-analysis, the results show that core training improves performance. They have found that participants in core-stability program were able to increase their throwing velocity by more than 5% [27]. Athletes who received strength training increased their throwing velocity by 6.9%. Throwing velocity was increased by 3.4% for participants in the CKCUES program [26,27,28,29]. It is comperable with the results of the present paper, where overall throwing accuracy was improved by 8% and throwing speed by 14.35%. A possible difference can be the duration of the program.

The results show there is an association between the trunk stability tests and shooting performance. It is conceivable that, by increasing the trunk stability, it enables better support for shoulder joint movements during the shooting movement, which contributes to an increase in shooting performance, which was theoretically mentioned in previous research too [12].

### 4.3. ER/IR Muscle Strength and Shooting Performance

The external rotator muscles exert pressure on the shoulder joint during movement, while playing a very important dynamic stabilizing role. The most likely position of injury is blocking or throwing [16,30,31,32,33]. During the shooting movement, a high-speed internal rotation movement is required, which has an exeptional importance in the acceleration phase of the throwing movement. They discovered that there is a relationship between traumatic injuries and internal rotator muscle weakness, which is explained by the difficulty of executing fast movements to avoid blocking attempts. Unlike the present paper, Bauer J. et al. (2020) examined the relationship between shoulder stability and the ER/IR ratio [16]. The Y-balance test showed a correlation with the ER/IR ratio [6] (0.06 ≤ r ≤ 0.34) as in the present study (r = 0.234; *p* = 0.004). Clarsen B. et al. (2014) found a link between increased shoulder problems and ER weakness [6]. Taking their results further, it was crucial to test an important stability determinant such as the ER/IR ratio to see if it had a relationship with recording performance. The present paper examined the correlation between ER/IR ratio and shooting performance, but it did not reach the significant level. Considering the dynamic stabilizing role of the rotator cuff muscles (mainly the supraspinatus) during throwing, as well as the role of the ER/IR muscle strength ratio in shoulder joint stability, it is likely that the isometric ER/IR muscle strength may have an indirect effect on the throwing performance, however the clarification needs further investigations.

### 4.4. Training Program

Raeder C. et al.’s (2015) results showed that a 6 week periodized Medicine Ball Throw Exercise Program (MBTEP) can significantly improve throwing velocity (with 14%) and shoulder external rotator muscle strength (with 10%) [19]. Similarly, a 6 weeks long performed Strength Training Program (STP) with elastic bands seemed also effective to improve external rotator strength and throwing speed for young female handball players, based on Mascarin N. C. et al.’s (2017) results [18]. The results in the present manuscript showed that handball players participating in the 12 weeks periodized training program experienced a significant improvement in overall throwing accuracy (with 8%) and throwing speed (with 14,35%).

From the results of the above-mentioned researches and the present paper, it can be concluded that by inducing sport-specific movement adaptations and creating proximal stability, it is possible to build an appropriate basis for the force transfer through the shoulder joint with the increased angular velocity characteristic of the throwing movement. Furthermore it can be interesting in determining the length of a program that a 6-week program can be just as effective in triggering adaptations as a longer, 12-week program.

## 5. Limitations

Due to geographical limitations, the researchers had to rely on the professionals working directly with the athletes and thus their engagement played a major role in ensuring compliance with the rules before baseline surveys. In addition, baseline surveys may have been confounded by other training completed by athlete in addition to the study protocol. The research team tried to minimize this bias by ensuring regular communication with the those coordinating the program and with the professionals working with the handball players and monitoring workload intensity. A randomized controlled trial is recommended for future studies.

Regarding the accuracy test, it may be worth doing further research on how it could be optimized for athletes of different heights, since the height of target and throwing position means a different angle in shoulder joint and scapula position and ER/IR muscle work too.

## 6. Future Directions and Practical Implications

### 6.1. Future Directions

Although the study of prospective shoulder injury was beyond the scope of the current study, given the association, further research investigating the relationship is warranted. Furthermore, it would be useful to conduct a follow-up starting at a young age, as athletes who have suffered a serious shoulder injury do not progress to premier-league championships. In the future, the development of an academic system will hopefully provide a suitable environment for this purpose. The effect of a sufficiently specific program could greatly contribute to reducing the number of shoulder injuries among handball players, a hypothesis that could be verified by further research.

### 6.2. Practical Implications

Using the Y-balance test can be a great help for physiotherapists working with overhead athletes in their daily work. Higher scores at the test have been associated with better results on other tests of shoulder joint stability from other aspects, for example ER muscle strength, and increased IR ROM, CKCUES test scores. It examines not only stability in a specific direction, but also in several directions in function, which also affects shooting performance. In order to be able to assess the shoulder joint stability and sport-specific performance of an athlete as comprehensively as possible, it is worth examining the performance of the Y-balance test and shooting performance tests in addition to the ROM, CKCUES, trunk stability and ER/IR muscle strength measurements, as the shoulder joint must not only be stable but also sufficiently mobile and functionally suitable for high exertion in handball.

## 7. Conclusions

The primary findings of this study provide support for the notion that trunk and shoulder stability play a crucial role in determining shooting performance among female handball players. While a causal relationship was not established, the study suggests that measuring and training, specifically focusing on medial shoulder stability, could be a beneficial addition to enhance shooting accuracy and speed in handball athletes.

## Figures and Tables

**Table 1:** Test reliability and validity.

	ICC (95% CI)	SEM_95_	MDC_95_
PROM IR °	0.96 (0.90–0.99)	2°	5°
PROM ER °	0.94 (0.82–0.98)	3°	7°
ER Strength (%)	0.87 (0.72–0.95)	0.37	0.52
IR Strength (%)	0.99 (0.97–0.99)	0.25	0.35
ER/IR Strength Ratio (%)	0.89 (0.74–0.95)	9.17	12.97
CKCUEST (touches)	0.96 (0.90–0.98)	2.76	3.91
YBT-UQ medial slide (cm)	0.94 (0.90–0.97)	2.9	8.1
YBT-UQ supero-lateral slide (cm)	0.96 (0.92–0.99)	2.3	6.4
YBT-UQ infero-lateral slide (cm)	0.92 (0.80–0.96)	2.2	6.1
Throwing accuracy	0.99 (0.99–1.00)	0.6	N/A
Throwing velocity	0.98 (0.89–0.99)	0.2	0.3

**Table 2:** Significant relationships of correlation calculations.

Test	Subtest	*p* Value	R Value
Y-balance medial stability	Throwing accuracy (right upper corner)	*p* < 0.001	R = 0.766
	Throwing accuracy (left upper corner)	*p* < 0.001	R = 0.729
	Overall throwing accuracy	*p* < 0.001	R = 0.907
	Trunk stability (4 point plank)	*p* = 0.001	R = 0.267
	Trunk stability (dominant side plank)	*p* = 0.002	R = 0.246
Y-balance supero-lateral stability	Throwing accuracy (right upper corner)	*p* < 0.001	R = 0.498
	Throwing accuracy (left upper corner)	*p* < 0.001	R = 0.634
	Overall throwing accuracy	*p* < 0.001	R = 0.694
	PROM (dominant shoulder IR)	*p* = 0.036	R = 0.171
	Trunk stability (4 point plank)	*p* = 0.027	R = 0.180
	Throwing velocity	*p* = 0.004	R = 0.231
Y-balance infero-lateral stability	Trunk stability (4 point plank)	*p* = 0.008	R = 0.215
	Trunk stability (dominant side plank)	*p* = 0.001	R = 0.276
	External rotator muscle strength	*p* = 0.004	R = 0.234
CKCUES test	Core-max test	*p* < 0.001	R = 0.301
	Trunk stability (dominant side plank)	*p* = 0.001	R = 0.258
	External rotator muscle strength	*p* = 0.003	R = 0.236
Trunk stability (4 point plank)	Throwing velocity	*p* = 0.008	R = 0.215
	Throwing accuracy (left upper corner)	*p* = 0.006	R = 0.223
	Overall throwing accuracy	*p* = 0.004	R = 0.234
Trunk stability (dominant side plank)	Throwing accuracy (right upper corner)	*p* = 0.027	R = 0.180
	Throwing accuracy (left upper corner)	*p* = 0.002	R = 0.247
	Overall throwing accuracy	*p* = 0.001	R = 0.261

**Table 3:** Descriptive statistics and differences between baseline and three-month measurements in Intervention Group (IG) and Control Group (CG).

	Intervention Group (IG)			Control Group (CG)		
	Before Training	After Training	Significance	Baseline	After Three Months	Significance
Dominant shoulder external rotation	111.78 ± 13.76°	102.17° ± 7.97°	*p* < 0.001	117.22 ± 8.26°	117.11 ± 5.75°	*p* = 0.97
Dominant shoulder internal rotation	58.47 ± 14.32°	68.11 ± 10.16°	*p* < 0.001	57.94 ± 18.32°	45.44 ± 9.20°	*p* = 0.02
Shoulder stability (CKCUES test)	100.22 ± 18.73 touches	117.17 ± 12.10 touches	*p* < 0.001	98.39 ± 31.85 touches	103.72 ± 15.61 touches	*p* = 0.49
Dominant shoulder anterior stability (YBT-UQ)	69.79 ± 12.49%	73.70 ± 11.69%	*p* < 0.001	62.09 ± 6.91%	75.67 ± 11.17%	*p* < 0.001
Dominant shoulder postero-lateral stability (YBT-UQ)	143.09 ± 14.94%	147.50 ± 13.49%	*p* < 0.001	91.61 ± 12.34%	98.63 ± 25.74%	*p* = 0.08
Dominant shoulder postero-medial stability (YBT-UQ)	107.80 ± 12.33%	110.04 ± 11.79%	*p* = 0.003	122.60 ± 16.70%	122.41 ± 20.74%	*p* = 0.98
Trunk stability (4 point plank)	118 ± 39.81 s	144 ± 33.64 s	*p* < 0.001	97.78 ± 34.96 s	173.28 ± 78.64 s	*p* = 0.002
Dominant shoulder external rotator muscle strength	15.96 ± 2.76 kg	19.77 ± 3.83 kg	*p* < 0.001	21.51 ± 6.51 kg	17.75 ± 2.80 kg	*p* < 0.01
Dominant shoulder external/internal rotator muscle strength rate between 60–72%	5 people	11 people	*p* = 0.055	2 people	3 people	*p* = 0.33
Trunk stability (Core-max test)	7.67 ± 1.65 levels	8.33 ± 0.91 levels	*p* = 0.004	6.11 ± 3.59 levels	7.88 ± 2.33 levels	*p* = 0.06
Trunk stability (dominant side plank)	80.2 ± 18.96 s	97.6 ± 15.98 s	*p* < 0.001	67.22 ± 29.82 s	100.22 ± 27.97 s	*p* = 0.01
Throwing velocity	15.47 ± 1.89 m/s	18.06 ± 2.55 m/s	*p* < 0.001	13.62 ± 1.18 m/s	14.52 ± 1.71 m/s	*p* = 0.08
Throwing accuracy (right upper corner)	3.78 ± 0.94 scores	3.89 ± 0.90 scores	*p* = 0.495	2.50 ± 1.38 scores	2.44 ± 1.34 scores	*p* = 0.83
Throwing accuracy (left upper corner)	3.50 ± 1.10 scores	4.00 ± 0.77 scores	*p* = 0.024	2.56 ± 1.42 scores	3.01 ± 1.35 scores	*p* = 0.08
Throwing accuracy (overall)	7.28 ± 1.49 scores	7.89 ± 1.37 scores	*p* = 0.002	5.06 ± 2.46 scores	5.5 ± 2.20 scores	*p* = 0.18

## Data Availability

Upon request, the corresponding author will provide the tables containing the data to the applicant.

## Supplementary Materials

The following supporting information can be downloaded at: https://www.mdpi.com/article/10.3390/sports12110296/s1, Appendix S1: Permission to conduct the research, Appendix S2: Description of the intervention protocol, Appendix S3: Schematic representation of the training protocol, Figure S1: Glenohumeral internal rotation deficit (GIRD), Figure S2: The Sleeper’s stretch position, Figure S3: The stretching of major pectoralis muscle (left) and the upper trapesoid muscle, Figure S4: Trunk and shoulder stabilization—side plank with Bosu, Figure S5: Trunk and shoulder stabilization on unstable surface with Fitball, Figure S6: The goal exercise of lower trapesoid muscle, Figure S7: External rotator strenghtening on stable surface—1. phase, Figure S8: External rotator strenghtening standin on dynair—2. phase, Figure S9: External rotator strenghtening on stable surface standing on one leg—3. phase, Figure S10: External rotator strengthening on dynair standing on one leg—4. phase, Figure S11: Serratus anterior muscle strengthening, Figure S12: Lower and transversal trapesiod muscle strengthening.
