# Supplementary material for: Effects of a Specific Trunk and Shoulder Strength Training Program on Throwing Velocity and Accuracy: A Study Among Hungarian First-League Female Handball Players
Source: Sports (Basel). 2024 Oct 31;12(11):296. doi: 10.3390/sports12110296 (PMC11598238; doi:10.3390/sports12110296)
Supplement: Supplementary file 1 [file sports-12-00296-s001.zip › sports-3047488-supplementary.pdf]

**INTERVENTION**

| Test | Description | Reference |
|---|---|---|
| **4 point plank**<br>**Side plank** | Start position: 4 point and side plank. Elbows under shoulder line, hand apart in shoulder width, feet apart hip width.<br>Instruction: Hold the position as long as it is possible, without any detectable posture change (trunk rotation, pelvic lifting). The result was recorded in seconds. The athlete was warned a maximum of three times if the position has changed compared to the start. The third warning was the end of the test. The test ended even if the athlete finished the plank herself, because she couldn't hold it any longer. One athlete was measured at a time. | [Nor Adnan N. M. et al, 2018] |
| **Core-max test** | Start position: high-plank<br>Instruction: Hold the position as long as it is possible, without any detectable posture change (trunk rotation, pelvic lifting). After 1 minute holding, there will be position change after 15 seconds, until the 9th level is completed. In case of loosing balance or touching the floor the test has ended and the number of the last successfully completed level has been registered.<br>Levels: can be seen in the attached figure<br>Level 1: 60 seconds high-plank<br>Level 2: 15 seconds right arm raised<br>Level 3: 15 seconds left arm raised<br>Level 4: 15 seconds right leg raised<br>Level 5: 15 seconds left leg raised<br>Level 6: 15 seconds left arm and right leg raised<br>Level 7: 15 seconds right arm and left leg raised<br>Level 8: 30 seconds high-plank not entirely completed<br>Level 9: 30 seconds high-plank completed | [Zemková E., 2022] |
| **Y-balance test** | Start position: high-plank<br>Instruction: Keep the high plank position with their feet shoulder wide apart and take the thumb of your stabile hand on the red line. From this, slide the bar to medial, supero-lateral and infero-lateral directions. The patient have to be able to come back from the end of the sliding without touching the floor or loosing balance. After every complete trial, a 30-s rest period was granted before completing the next trial. | [Bauer J. et al, 2020]<br>[Gorman P. P. et al, 2012]<br>[Tucci H. T. et al, 2014] |
| **CKCUES test** | Start position: high-plank<br>Instruction: Keep the position without any detectable posture change (trunk rotation, pelvic lifting) for 15 seconds and touch the opposite shoulder with your hand alternately as many times as it is possible.<br>Normal rate: >21 | [Tucci H. T. et al, 2014] |
| **PROM** | Start position: supine<br>Description: passive shoulder external and internal rotational range of movement was measured using a goniometer in a 90° abducted and neutrally rotated shoulder position and a bent elbow with the scapula fixed. It was taken to the end range. | [Clarsen B. et al, 2014]<br>[Vigolvino L. P. et al, 2019] |

| | | |
|---|---|---|
| **ER/IR strength** | Start position: supine<br>Device setting: at the most distal part of the forearm, in the area of wrist<br>Description: with the participant in supine position their shoulder in 90° abducted and neutrally rotated the strength of external and internal rotator muscles was measured during an isometric contraction. ER muscles strength was measured in the middle of the ER ROM, while IR muscles strength was measured in the middle of the IR ROM. We verbally and manually assisted players to stabilise their scapula. During the measurment patients were asked to performe the maximal strength in each direction. | [Clarsen B. et al, 2014]<br>[Vigolvino L. P. et al, 2019] |
| **Shooting velocity** | Start position: 7 meters far from the goal line and set in the middle of the goal<br>Instruction: At the whistle, the player must shoot the ball into the center of the goal as hard as she can. The ball MUST NOT touch the ground in front of the goal line. The players had to use a throwing technique with the elbow at or above shoulder height.<br>Description: To calculate the velocity the video analysis was performed with the Kinovea system, which used the formula $v = s / t$. The female athletes used a standard ball size 1. | [Nor Adnan N. M. et al, 2018]<br>[Bauer J. et al, 2020] |
| **Shooting accuracy** | Start position: 7 meters far from the goal line and set in the middle of the goal<br>Instruction: At a whistle, randomly (staff member announced which corner), the handball players had to score as many times as possible out of ten shots into a triangle measured in the upper right and left corners of the cage. The players had to use a throwing technique with the elbow at or above shoulder height.<br>Description: The triangles were 60 cm long horizontally, 40 cm long vertically and 72 cm long diagonally, and adjusted with rubber spiders. The female athletes used a standard ball size 1. | [Bauer J. et al, 2020] |

***Appendix S2:*** Description of the intervention protocol

**TRAINING PROGRAM**

A three-month, one-hour physiotherapy program was performed twice a week, based on strengthening the periscapular and shoulder stabilizing muscles, the trunk stabilizing muscles of the players on a stable surface during the first month to create proximal stability for distal mobility. The trainings ended with twenty minutes long stretching session each time, in which contract-relax and static stretching exercises were performed.

| | Sets x Repetitions x Load | Rest (s) | Sets x Repetitions x Time (s) | Rest (s) |
|---|---|---|---|---|

| | | | | |
|---|---|---|---|---|
| **Week 1-2** | 2 x 12 x AW<br>-lower and transversal trapesoid muscle strengthening (Figure S12) | 60 between sets<br>5 between repetitions | 1 x 4 x 20 (each side)<br>- Sleeper’s stretch static stretching<br><br>1 x 2 x 20<br>- Upper trapesoid muscle static stretching (Figure S3) | 15 between sets |
| | 3 x 30 sec x BW<br>- supine plank | 30 between sets | | |
| | 4 x 30 sec x BW/side<br>supine bridgeing position with one leg (other leg is in the air 45° abducted) | 30 between sets | | |
| | 3 x 10 x BW<br>- pelvic lifting, legs extend alternately | 30 between sets | | |
| | 3 x 30 sec x BW<br>- side plank with knee support | 30 between sets<br>5 between repetitions | | |
| | 3 x 8 x 3 RB<br>- serratus anterior muscle strengthening (Figure S11) | 30 between sets | | |
| | 4 x 30 sec x BW<br>- 4 point plank with both legs on bosu | 30 between sets | | |
| | 4 x 30 sec x BW/ side<br>- side lying with abducted legs, lower leg tucked into TRX rope | 30 between sets | | |

| | | | | |
|---|---|---|---|---|
| **Week 3-4** | 3 x 12 x 1<br>- WXY in prone position | 60 between sets<br>5 between repetitions | 1 x 4 x 20 (each side)<br>- Sleeper’s stretch static stretching | 15 between sets |
| | 4 x 30 sec x BW/side<br>supine bridgeing position with one leg in TRX (other leg is in the air 45° abducted) | 15 between repetitions | 1 x 2 x 20<br>- Major pectoral muscle static stretching (Figure S3) | |
| | 3 x 30 sec x BW<br>- supine plank legs on Bosu | 30 between sets | | |
| | 2 x 12 x BW/side<br>- supine plank legs on bosu, alternately lift up one leg, thank change it continously | 30 between sets | | |
| | 3 x 10 x BW<br>- pelvic lifting, one leg extend alternately on Bosu | 30 between sets | | |
| | 3 x 30 sec x BW<br>- side plankwith knee support | 30 between sets | | |
| | 3 x 10 x 4 RB<br>- serratus anterior muscle strengthening | 30 between sets<br>5 between repetitions | | |
| | 4 x 30 sec x BW/side<br>- 4 point plank with one leg on bosu | 30 between sets | | |
| | 4 x 10 x BW<br>- 4 point plank with one leg on bosu, alternately lift up one leg, thank change it continously | 30 between sets | | |
| | 4 x 30 sec x BW/ side<br>- side lying with abducted legs, lower leg tucked into TRX rope | 30 between sets | | |

| | | | | |
|---|---|---|---|---|
| **Week 5-6** | 2 x 20 x 1,5 kg<br>- WXY in prone position, laying on a fitball | 30 between sets<br>5 between repetitions | 1 x 4 x 20 (each side)<br>- Sleeper's stretch static stretching | 15 between sets |
| | 4 x 45 sec x BW/side supine bridgeing position with one leg in TRX (other leg is in the air 45° abducted). Softball under thoracic spine, elbow support | 30 between sets | 1 x 2 x 20<br>- Upper trapesoid muscle static stretching (Figure S3) | |
| | 2 x 12 x BW/side<br>- supine plank legs on bosu, alternately lift up one leg, thank change it continously, arms crossed in front of the body | 30 between sets | 1 x 2 x 20<br>- Major pectoral muscle static stretching (Figure S3) | |
| | 4 x 10 x BW/side<br>- pelvic lifting, one leg extend alternately on Bosu | 30 between sets | | |
| | 3 x 10 x BW<br>- serratus anterior muscle strengthening in all fours | 30 between sets<br>5 between repetitions | | |
| | 4 x 45 sec x BW<br>- stabile crunch plank with two legs in TRX rope | 30 between sets | | |
| | 4 x 12 x BW<br>- dynamic crunch plank with two legs in TRX rope, pull in with knees | 30 between sets | | |
| | 4 x 10 x BW<br>- 4 point plank with one leg on bosu, alternately lift up one leg, thank change it continously | 30 between sets | | |
| | 4 x 30 sec x BW/ side<br>- side lying with abducted legs, lower leg tucked into | 30 between sets | | |

| | | | | |
|---|---|---|---|---|
| | TRX rope, dinair under the supporting shoulder<br><br>2 x 20 x BW<br>- deadbug exercise<br><br>2 x 10 x 3 RB<br>- Reverse throwing movement, kneeling on a Bosu | <br><br><br>30 between sets<br><br>30 between sets | | |
| **Week 7-8** | 2 x 20 x 2<br>- WXY in prone position<br><br>4 x 45 sec x BW/side supine bridgeing position with one leg on fitball (other leg is in the air 45° abducted), elbow support<br><br>2 x 12 x BW/side<br>- supine plank legs on fitball, alternately lift up one leg, thank change it continously, arms crossed in front of the body<br><br>4 x 45 sec x BW<br>- plank on fitball<br><br>2 x 10 x BW/side<br>- plank on fitball, alternately lift up one leg, thank change it continously<br><br>3 x 10 x 3 RB<br>- serratus anterior muscle strengthening in all fours<br><br>3 x 45 sec x BW/side<br>- stabile crunch plank with one leg in TRX rope<br><br>3 x 12 x BW/side | 30 between sets<br>5 between repetitions<br><br>30 between sets<br>5 between repetitions<br><br>30 between sets<br><br>30 between sets<br><br>30 between sets<br><br>30 between sets<br><br>30 between sets<br><br>30 between sets | 1 x 4 (each side)<br>- Sleeper's stretch contract-relax stretching<br><br>1 x 2 x 20<br>- Upper trapesoid muscle static stretching (Figure S3)<br><br>1 x 2<br>- Major pectoral muscle contract-relax stretching (Figure S3) | 15 between sets |

| | | | | |
|---|---|---|---|---|
| | - dynamic crunch plank with one leg in TRX rope, pull in with knees | | | |
| | 4 x 45 sec x BW/ side<br>- side lying with abducted legs, lower leg tucked into TRX rope, dinair under the supporting shoulder | 30 between sets | | |
| | 2 x 20 x BW<br>- deadbug exercise | 30 between sets | | |
| | 2 x 10 x 3 RB<br>- Reverse throwing movement, kneeling on a Bosu | 30 between sets | | |
| | 4 x 10 x BW<br>- TRX push up with knee support | 30 between sets | | |
| | 2 x 10 x BW<br>- lower trapesoid strenghtening exercise (Figure S6) | 30 between sets | | |
| | 3 x 10 x 5 RB<br>- serratus anterior muscle strengthening in all fours | 30 between sets | | |
| | 2 x 12 x 2 RB<br>- Plyometric throwing two leg stand on stabile surface (Figure S7)<br>- Plyometric throwing two leg stand on unstable surface (Figure S8) | 30 between sets | | |
| **Week 9-10** | 2 x 30 x 1 BB<br>- Perturbation on throwing and nonthrowing hand | 30 between sets | 1 x 4 (each side)<br>- Sleeper’s stretch contract-relax stretching | 15 between sets |
| | 4 x 45 sec x BW/side supine bridgeing position with one leg on fitball | 30 between sets | 1 x 2 x 20 | |

| | | | |
|---|---|---|---|
| | (other leg is in the air 45° abducted), elbow support | | - Upper trapesoid muscle static stretching (Figure S3) |
| | 4 x 60 sec x BW<br>- plank on fitball | 30 between sets | 1 x 2<br>- Major pectoral muscle contract-relax stretching (Figure S3) |
| | 3 x 45 sec x BW/side<br>- stabile crunch plank with one leg on fitball (other leg is 45°abducted) | 30 between sets | |
| | 3 x 12 x BW/side<br>- dynamic crunch plank with one leg on fitball (other leg 45°abducted), pull in with knees | 30 between sets | |
| | 2 x 10 x BW<br>- Ball rolling forward and backward with hands (Figure S5) | 30 between sets | |
| | 4 x 12 x BW<br>- TRX push up | 30 between sets | |
| | 3 x 10 x BW<br>- Lower trapesoid strengthening in hanging position (Figure S6) | 30 between sets | |
| | 4 x 45 sec x BW/ side<br>- side lying with abducted legs, lower leg tucked into TRX rope, dinair under the supporting shoulder | 30 between sets | |
| | 2 x 12 x BW<br>- Superman with TRX rope with knee support | 30 between sets | |
| | 2 x 10 x 1<br>- Plyometric ball throw in all fours position (external rotation) | 30 between sets | |
| | 2 x 12 x 2 RB | | |

| | | | | |
|---|---|---|---|---|
| | - Plyometric throwing one leg stand on stabile surface (Figure S9)<br>- Plyometric throwing one leg stand on unstable surface (Figure S10) | 30 between sets | | |
| **Week 11-12** | 2 x 40 x 1,5 BB<br>- Perturbation on throwing and non-throwing hand<br><br>4 x 12 x BW<br>- Side plank, trunk rotation with 1 kg BB in hands, legs on Bosu (Figure S4)<br>4 x 12 x BW<br>- Lower trapesoid strengthening in hanging position (Figure S6)<br><br>3 x 45 sec x BW/side<br>- stabile crunch plank with one leg on fitball (other leg is 45°abducted)<br><br>4 x 12 x BW/side<br>- dynamic crunch plank with one leg on fitball (other leg 45°abducted), pull in with knees<br><br>4 x 10 x BW<br>- Ball rolling forward and backward with hands (Figure S5)<br><br>4 x 20 x BW/side<br>- mountain running exercise in high-plank position, hands gripping the side of a bosu<br><br>4 x 12 x BW<br>- TRX push up one leg lifted<br><br>4 x 60 sec x BW/ side | 30 between sets<br><br>30 between sets<br><br>30 between sets<br><br>30 between sets<br><br>30 between sets<br><br>30 between sets<br><br>30 between sets<br><br>30 between sets<br><br>30 between sets | 1 x 4 (each side)<br>- Sleeper's stretch contract-relax stretching<br><br>1 x 2 x 20<br>- Upper trapesoid muscle static stretching (Figure S3)<br><br>1 x 2<br>- Major pectoral muscle contract-relax stretching (Figure S3) | 15 between sets |

| | | |
|---|---|---|
| | - side lying with abducted legs, lower leg tucked into TRX rope, dinair under the supporting shoulder | |
| | 4 x 12 x BW | 30 between sets |
| | - Superman with TRX rope | |
| | 2 x 10 x 2 | 30 between sets |
| | - Plyometric ball throw in all fours position (external rotation) | |
| | 2 x 12 x 3RB | 30 between sets |
| | - Plyometric throwing one leg stands on stabile surface (Figure S9)<br>- Plyometric throwing one leg stands on unstable surface (Figure S10) | |

***Appendix S3:*** Schematic representation of the training protocol

Abbreviations: Body weight (BW), Rubber band (RB), Barbell (BB), Arm weight (AW), Ball weight (BallW)

**SZENT IMRE KÓRHÁZ és SZENT JÁNOS KÓRHÁZ**
**Közös Regionális Tudományos és Kutatásetikai Bizottsága**
**Elnök: Dr. Finta Ervin Ph.D.**
e-mail: ikeb@szentimrekorhaz.hu
1115 Budapest, Tétényi út 12-16. ✉ 1502 Budapest, Pf. 4. – ☎+361-464-8600/1771 – Fax:+361-203-3645

A Tanúsítvány regisztrációs száma: 01-18068/18-12978,-12979

**JEGYZŐKÖNYV** **18/2019**

**A vizsgálat címe:** "Kézilabdázók sérüléseinek felmérése, sérülésregiszter felállítása, a hajlamosító tényezők meghatározása"
**A vizsgálatot kérelmező cég:** Országos Sportegészségügyi Intézet Sportsebészeti Osztály
**A vizsgálat vezetője:** Dr.Pavlik Attila ortopéd sebész, sportszakorvos
**Független orvos:** Dr. Kolossváry Endre
**Benyújtott dokumentumok, és az elbíráláshoz szükséges adatok**

- Vizsgálati terv magyar nyelvű összefoglalója
- Magyar nyelvű betegtájékoztató és beteg beleegyező nyilatkozat

**A vizsgálat célja:** tudományos kutatás
**A vizsgálat fázisbesorolása:** tudományos kutatás
**A bizottság jelenlévő tagjai:**

| | |
|---|---|
| Dr. Finta Ervin | főorvos a Bizottság elnöke |
| Dr. Kolossváry Endre | főorvos a Bizottság titkára |
| Prof Dr. Járai Zoltán | főorvos |
| Csák Annamária | vezetőápoló |
| Dr. Ferencz Mária | főorvos |
| Dr. Szabó Anita | főgyógyszerész |
| Dr. Hornyik Hajnalka | Jogi Iroda |
| Bulkai Katalin | vegyészmérnök |
| Dr. Tápay Tibor | nyugalmazott állatorvos |

**A bizottság döntése:**
A Bizottság a benyújtott dokumentációt áttekintette. A vizsgálat személyi és tárgyi feltételei adottak, kutatásetikai szempontból kifogásolhatót nem talált. Tudomásul vétel.
Kérjük a vizsgálat megkezdéséről az IKEB-et értesíteni szíveskedjenek, mert a bizottság figyelemmel kíséri a vizsgálat lefolytatását.
A Bizottság a 35/2005 (VIII.26) Eü.Miniszteri rendeletben előírtaknak megfelelően működik és működési szabályzata megfelel az ICH-GCP követelményeinek.

Budapest, 2019. december 5.

Dr. Finta Ervin
IKEB elnök

*Appendix S1:* Permission to conduct research

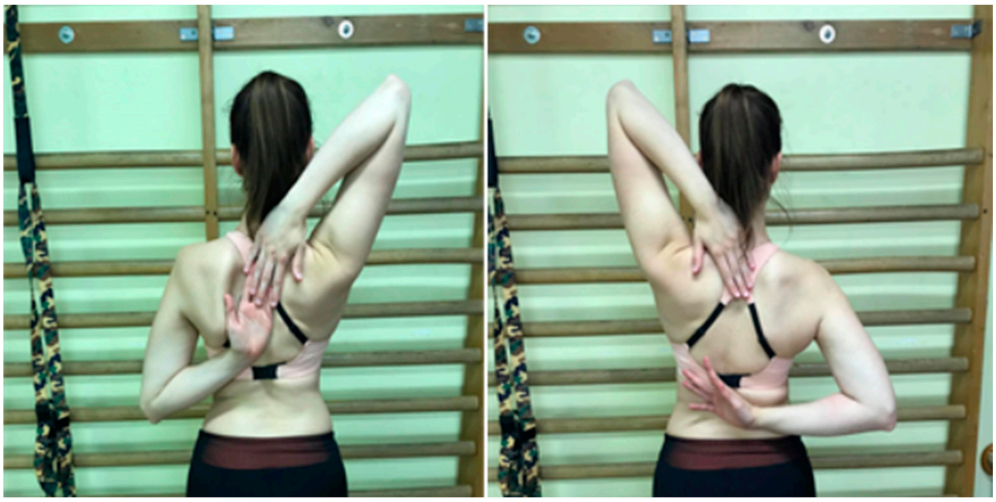

*Figure S1:* Glenohumeral internal rotation deficit (GIRD)

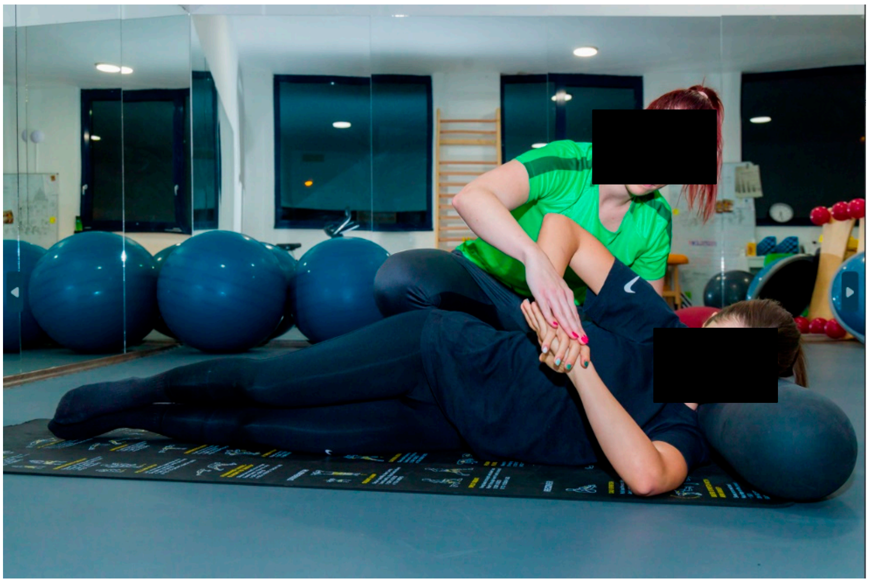

*Figure S2:* The Sleeper's stretch position

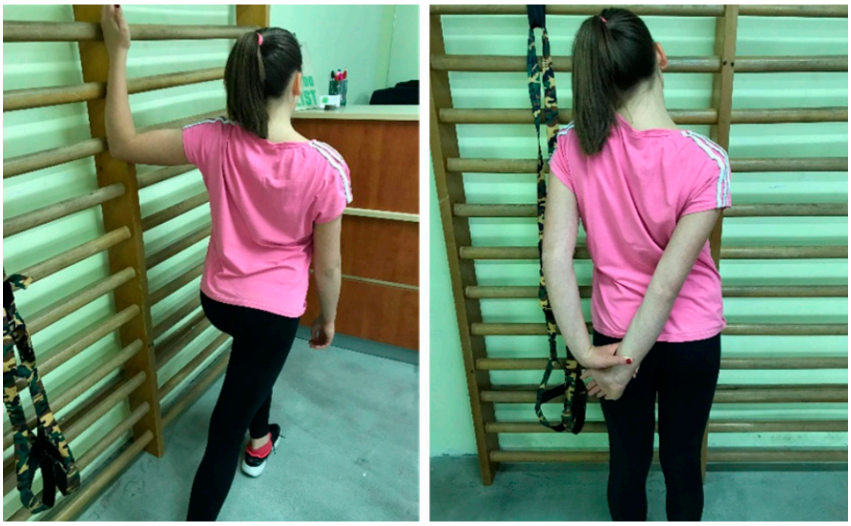

*Figure S3:* The stretching of major pectoralis muscle (left) and the upper trapesoid muscle

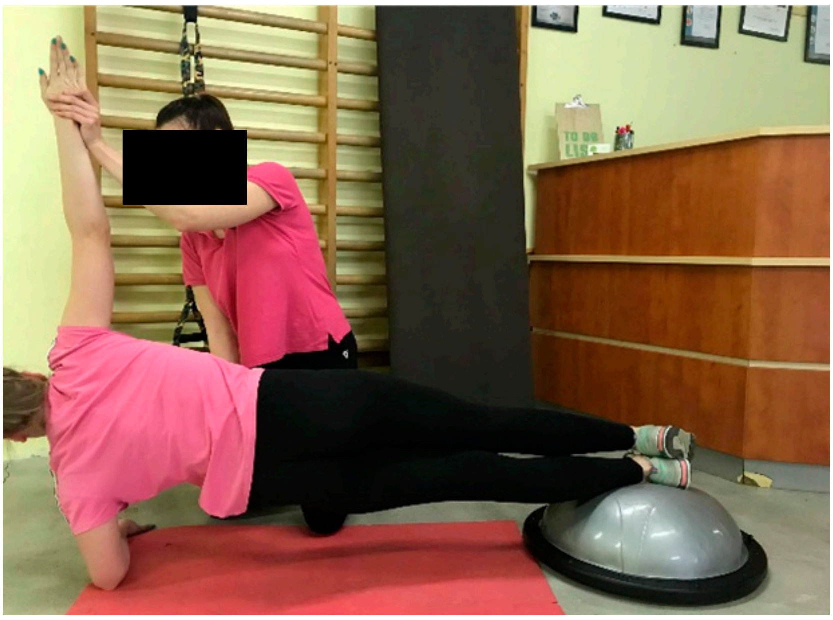

*Figure S4:* Trunk and shoulder stabilization – side plank with Bosu

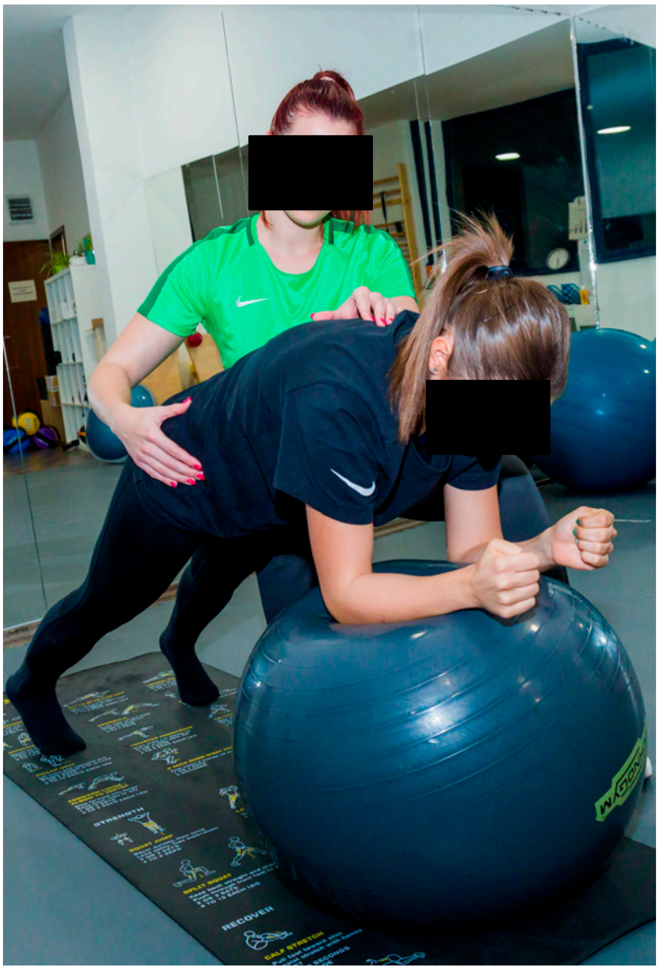

*Figure S5:* Trunk and shoulder stabilization on unstable surface with Fitball

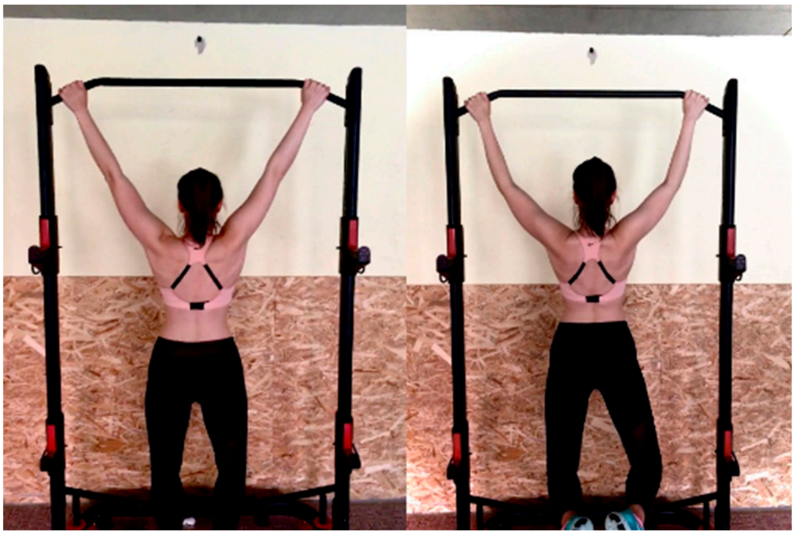

Figure S6: The goal exercise of lower trapesoid muscle

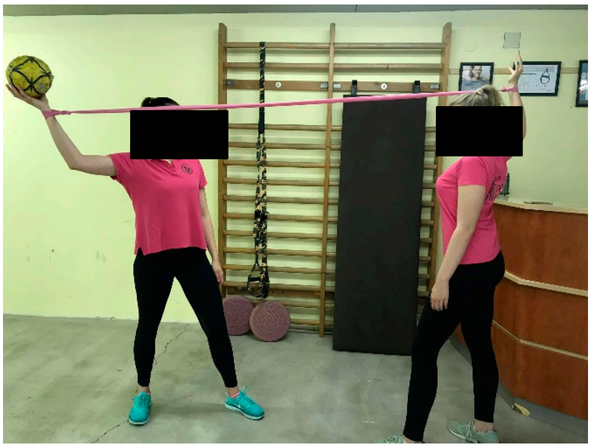

*Figure S7:* External rotator strenghtening on stable surface - 1. phase

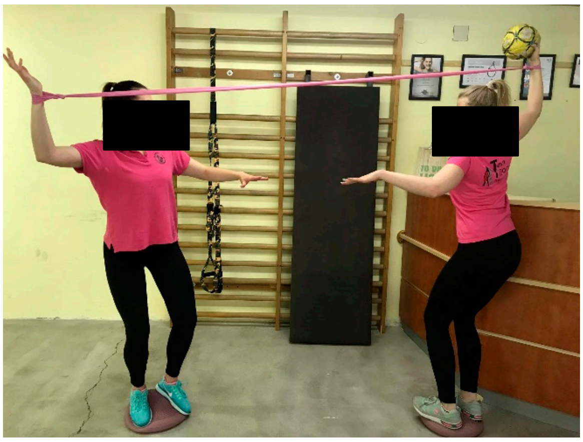

*Figure S8:* External rotator strenghtening standin on dynair - 2. phase

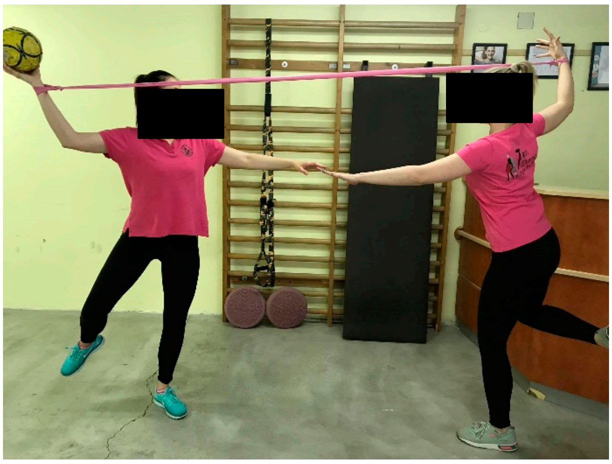

*Figure S9:* External rotator strenghtening on stable surface standing on one leg - 3. phase

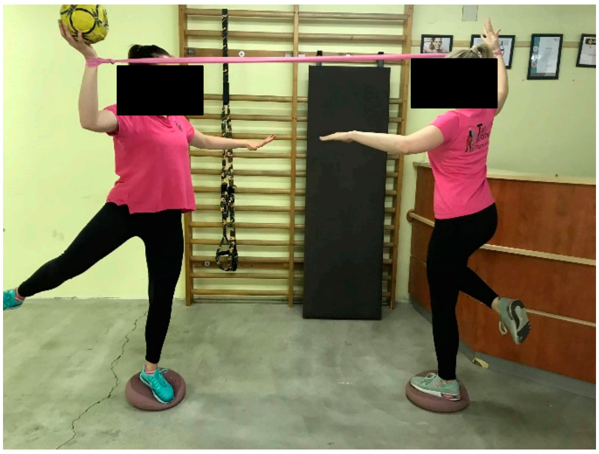

*Figure S10:* External rotator strengthening on dynair standing on one leg - 4. phase

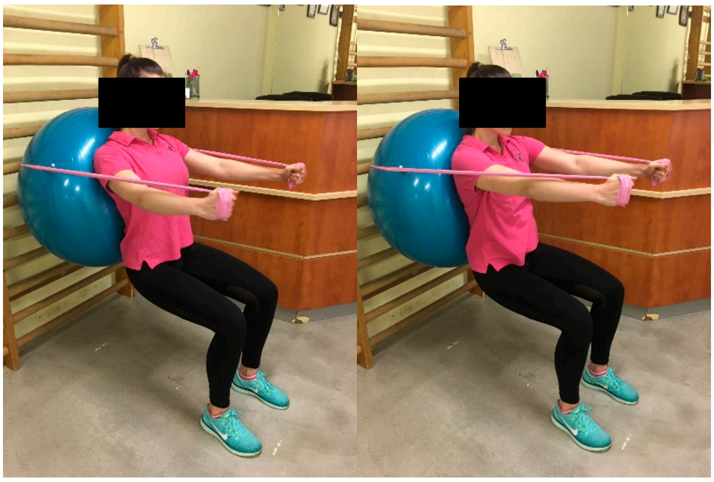

*Figure S11:* Serratus anterior muscle strengthening

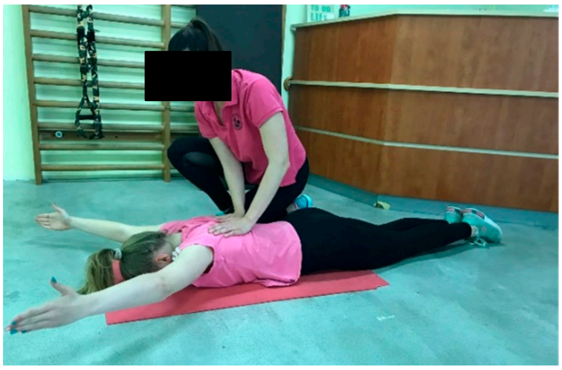

*Figure S12:* Lower and transversal trapesiod muscle strengthening
